# Time-restricted feeding downregulates cholesterol biosynthesis program via RORγ-mediated chromatin modification in porcine liver organoids

**DOI:** 10.1186/s40104-020-00511-9

**Published:** 2020-11-02

**Authors:** Kexin Zhang, Hao Li, Zimeng Xin, Yanwei Li, Xiaolong Wang, Yun Hu, Haoyu Liu, Demin Cai

**Affiliations:** 1 College of Animal Science and Technology, Yangzhou University, Yangzhou, 225009 PR China; 2 Institute of Epigenetics and Epigenomics, Yangzhou University, Yangzhou, 225009 PR China

**Keywords:** Cholesterol biosynthesis program, Histone modification, Pig, Porcine liver organoids, RORγ, Time-restricted feeding

## Abstract

**Background:**

Time-restricted feeding (TRF) is a dieting strategy based on nutrients availability and diurnal rhythm, shown to improve lipid metabolism efficiency. We have demonstrated previously that retinoic acid-related (RAR) orphan receptor (ROR) γ is the primary transcription factor controlling cholesterol (CHO) biosynthesis program of animals. However, the functional role of RORγ in liver physiology of pigs in response to TRF has not been determined, largely due to the lack of functional models and molecular tools. In the present study, we established porcine liver organoids and subjected them to restricted nutrients supply for 10-h during the light portion of the day.

**Results:**

Our results showed that TRF regimen did not alter hepatocyte physiology, including unchanged cell viability, caspase 3/7 enzyme activity and the gene signature of cell proliferation in porcine liver organoids, compared to the control group (*P* > 0.05). Furthermore, we found that TRF downregulated the hepatic CHO biosynthesis program at both mRNA and protein levels, along with the reduced cellular CHO content in porcine liver organoids (*P* < 0.05). Using unbiased bioinformatic analysis of a previous ChIP-seq data and ChIP-qPCR validation, we revealed RORγ as the predominant transcription factor that responded to TRF, amongst the 12 targeted nuclear receptors (NRs) (*P* < 0.05). This was likely through RORγ direct binding to the MVK gene (encoding mevalonate kinase). Finally, we showed that RORγ agonists and overexpression enhanced the enrichment of co-factor p300, histone marks H3K27ac and H3K4me1/2, as well as RNA polymerase II (Pol-II) at the locus of MVK, in TRF-porcine liver organoids, compared to TRF-vector control (*P* < 0.05).

**Conclusions:**

Our findings demonstrate that TRF triggers the RORγ-mediated chromatin remodeling at the locus of CHO biosynthesis genes in porcine liver organoids and further improves lipid metabolism.

## Background

Obesity is a major risk factor for chronic disorders such as nonalcoholic fatty liver disease (NAFLD), cardiovascular disease, and type II diabetes [1]. The etiology of obesity is complex, including nutrient imbalance and the disruption of multiple metabolic pathways in the liver [2, 3]. In addition to the dysregulation of glucose, lipid and cholesterol metabolism, it has been suggested that circadian rhythm is a major contributor to the pathophysiology of obesity [4–6]. The circadian rhythm is an evolutionarily conserved system in mammals that coordinates rhythms of behavior and physiology in response to predictable environmental changes in a near 24-h solar day [7, 8]. Although the circadian clock is a ‘build-in’ system, it is entrained to the local environment by external cues, including light, temperature and feeding time [4, 5].

Time-restricted feeding (TRF), a defined daily period of feeding and fasting [9, 10], is increasingly recognized as a preventative intervention against nutritional challenges in animals and humans [4, 6]. Studies reported that TRF reduces fat depot and weight gain in mice under high-fat feeding and ameliorates metabolic disorders [11, 12]. It is reported that TRF can also reduce serum cholesterol (CHO) levels in obese mice [6, 11, 12]. Furthermore, TRF downregulates the master lipid regulator peroxisome proliferator-activated receptorα and enzymes involved in triglyceride metabolism in the liver [4], as well as controls hepatic transcriptome in both wild type and the clock-disrupted mice [8]. Remarkably, it has been demonstrated that around 10–15% of all liver mRNA are expressed in a rhythmic fashion. Many of these genes play a role in cholesterol and glucose metabolism [4, 5, 8] with one essential part being retinoic acid-related (RAR) orphan receptor (ROR) [4, 13, 14]. Both RORγ and RORα are involved in controlling hepatic circadian rhythmic expression of glucose genes, whereas mice deficient in RORγ showed improved insulin sensitivity and glucose tolerance, especially at daytime [14]. In addition, it is recently reported that RORγ dictates the entire CHO biosynthesis pathway in cancerous cells and overrides the classic transcription factor sterol regulatory element-binding protein (SREBP)-2 [13]. However, the role of RORγ in cholesterol biosynthesis in liver physiology of mammals and its relation to TRF remain unclear.

To date, most of the TRF studies were carried out using mice or Drosophila [5, 15, 16]. As light cycle impacts animal circadian phenotype, it is noteworthy that mice are nocturnal, in contrast to diurnal mammals such as humans and pigs [17]. In this regard, pigs (*Sus scrofa*) are increasingly used as an animal model since they share anatomical, physiological, and immunological similarities with human beings [18, 19]. Herein we developed a porcine liver organoid model, which combines porcine traits and the ease of genetic manipulation in basic and pharmacological research, as well as to evaluate dieting strategy in livestock management.

Indeed, liver organoid culture is becoming a popular alternative of primary cell culture to recapitulate tissues in a dish [20, 21] and to study liver physiology and disease pathogenesis in human and mice [22]. Using extracellular matrix (Matrigel), the unique system enables organoids to resemble architectural and functional properties of *in vivo* tissue more closely [22], though the establishment using 3D culture could affect cell proliferation, morphogenesis and survival [23]. Nevertheless, such an approach allows the removal of confounding effects and provides a reductionist model of *in vivo* tissue [20, 22], yet not well-established in large animals.

In the current study, the effects of 10-h TRF on liver tissues were investigated using transcriptomics and chromatin immunoprecipitation, by applying temporal regulation of feeding cells nutrients in porcine liver organoids *in vitro.* We hypothesized that under normal healthy condition, TRF modifies the cyclical expression of metabolic regulators and associated cellular processes, thus improves metabolism.

## Methods

### Animals and the porcine liver organoid establishment

All animal procedures were in line with and approved by the Animal Ethical Committee of Yangzhou University (NSFC2020-DKXY-20). Liver tissues were obtained from 3 days old male piglets. Porcine organoids were established and cultured as previously described with modifications [24]. Briefly, dissected liver tissues of newborn piglet were finely minced and transferred to a 50-mL conical tube including a digestion mixture consisting of serum-free DMEM/F-12 medium (Gibco, basal medium) and 2.5 mg/mL collagenase D (Sigma), and were incubated for 1 h at 37 °C. Single cells were collected and mixed with 50 μL of Matrigel (BD Biosciences) and seeded in 24-well plates (Greiner bio-one) at a density of 1000 per well. When the matrix was solidified, 500 μL isolation medium (1:50 B27 supplement without vitamin A), 1:100 N2 supplement, 1 mmol/L N-acetylcysteine, 10% (vol/vol) Rspo1-conditioned medium, 10 mmol/L nicotinamide, 10 nmol/L recombinant human [Leu_15_]-gastrin I, 50 ng/mL recombinant human EGF, 100 ng/mL recombinant human FGF10, 25 ng/mL recombinant human HGF, 10 μmol/L Forskolin and 5 μmol/L A83-01, 25 ng/mL recombinant human Noggin or 5% (vol/vol) Noggin-conditioned medium, 30% (vol/vol) Wnt3a-conditioned medium and 10 μmol/L Rho kinase (ROCK) inhibitor were incubated for 4 d. Then the medium was replaced with normal liver expansion medium (1:50 B27 supplement without vitamin A, 1:100 N2 supplement, 1 mmol/L N-acetylcysteine, 10% (vol/vol) Rspo1-conditioned medium, 10 mmol/L nicotinamide, 10 nmol/L recombinant human [Leu^15^] -gastrin I, 50 ng/mL recombinant human EGF, 100 ng/mL recombinant human FGF10, 25 ng/mL recombinant human HGF, 10 μmol/L Forskolin and 5 μmol/L A83-01). The medium was changed every 3–4 d.

### Dexamethasone synchronization and sample collection

**Fig. 1:**
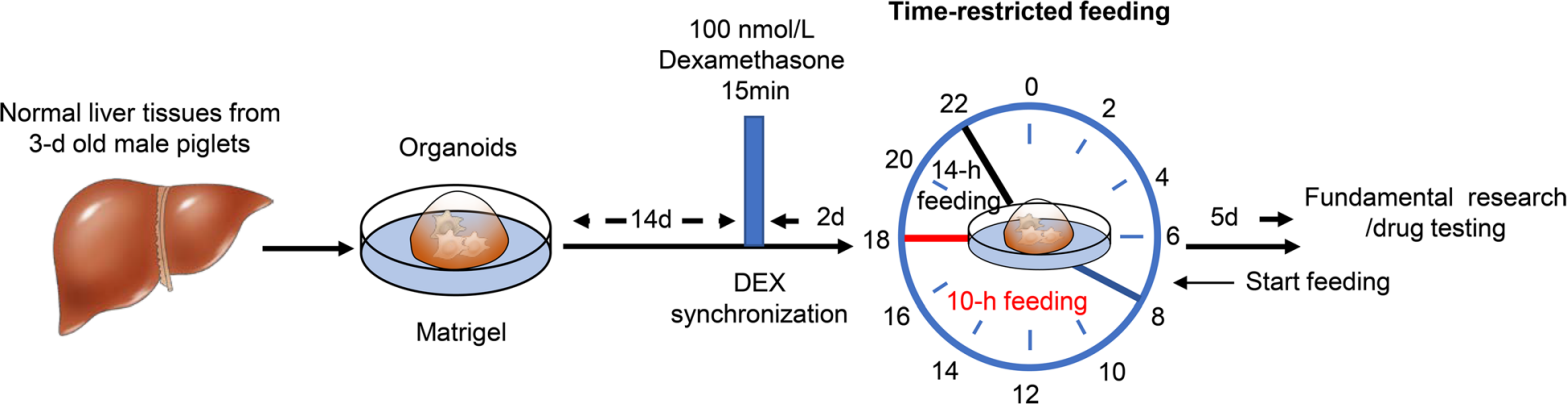
Illustration of the experimental design. The porcine liver organoids are derived from biopsies of normal liver tissues that can be used as a model in basic research and drug testing. The organoids were placed in Matrigel with the optimized media for 14 days after seeding and were treated with dexamethasone (DEX) for synchronization. They were subjected to the 24-h cycle of time-restricted feeding for 7 consecutive days: for the control group, the organoids were exposed to expansion medium for 14 h from 8:00 to 22:00 and to basal medium for 10 h from 22:00 to 8:00(+1 d); For the TRF group, the exposure to expansion medium was restricted to 10 h from 8:00 to 18:00 and to basal medium for 14 h from 18:00 to 8:00 (+1 d)

At day 15 from seeding, organoids of 12 wells were treated with 100 nmol/L (final concentration) of dexamethasone (DEX, Sigma-Aldrich) for 15 min to synchronize. The organoids were then washed three times with PBS (37 °C) and were incubated in expansion medium. Forty-eight hours after DEX treatment, organoids of 6 wells as control group were exposed to expansion medium for 14 h from 8:00 to 22:00 and to basal medium for 10 h from 22:00 to 8:00 (+1 d) in a 24-h cycle. Whereas organoids of the other 6 wells as TRF group were exposed to expansion medium for 10 h from 8:00 to 18:00 and to basal medium for 14 h from 18:00 to 8:00 (+1 d) in a 24-h cycle (Fig. 1). The exposure in the pattern of 24-h cycle was continued to 7 d and the organoids were harvested directly at 8:00 for fundamental testing. For the compounds/lentivirus treatment, the organoids were treated at 8:00 from the end of the 7^th^ day of 24-h cycle for another 48-h period and then harvested for measurements.

### Cell viability and caspase 3/7 activity in organoids

The organoids were seeded in 96-well plates at the density of 100 organoids in 10 μL Matrigel per well in a total volume of 100 μL expansion medium for 7 d exposure of the above 14/10 h feeding window and incubated with expansion medium for 48 h. Carefully aspirating the medium and adding 100 μL live/dead reagents (Thermofisher Scientific) for 30 min incubation at room temperature. Fluorescence microscopy was used to capture signals of a cell-permeant dye Calcein AM that represents live cells, and signals of ethidium bromide homodimer-1 to identify dead cells. Besides, Cell-Titer GLO reagents (Promega) were added and luminescence was measured on GLOMAX microplate luminometer (Promega) according to the manufacturer’s instructions. The caspase-3/7 activity was determined using a luminescent caspase-Glo 3/7 assay kit (Promega Corporation, Madison, USA) following the manufacturer’s instructions. The above assays were performed in triplicates and the entire experiments were repeated three times.

### qRT-PCR and western blotting analysis

Total RNA of 2 μg was isolated from organoids in 24-well plates, and the cDNA was prepared, amplified and measured using SYBR green as previously described [13]. Briefly, the fluorescent values were collected, and a melting curve analysis was performed. Fold difference was calculated [13]. The primers are shown in Table S1. The experiments were performed at least three times with data presented as mean values ± SD. Organoids lysates were analyzed by western blotting with antibodies specifically recognizing the indicated proteins shown in Table S2.

### Ectopic lentivirus production

For RORγ overexpression, porcine RORγ cDNA in pLX304 (DNASU) was amplified and cloned into a modified pLX304 vector as previously described [13]. Lentiviral particles were produced in 293 T cells after co-transfection of the above lentivirus vectors, psPAX2 and pMD2.G in 10-cm dishes.

### Measurement of cholesterol contents

Organoids were washed three times with cold PBS and subjected to extraction with organic solvents (7:11:0.1, chloroform/isopropanol/Triton X-100). Free (3-OH) and total (3-OH and esters) sterol levels were measured using Amplex™ Red Cholesterol Assay Kit (Thermofisher Scientific) and normalized to protein concentrations. All experimental points were set up as sextuplicate as biological replication and the entire experiments were repeated three times.

### ChIP-qPCR analysis and ChIP-seq data analysis

Briefly, organoids of 24-well plates were pelleted in cold PBS and resuspended in fixing buffer (50 mmol/L Hepes-KOH, 100 mmol/L NaCl, 1 mmol/L EDTA, 0.5 mol/L EGTA) before subject to crosslinking in 1% formaldehyde for 5 min followed by quenching with glycine for 5 min on ice. The pellets were collected by centrifugation and resuspended in lysis buffer (50 mmol/L HEPES pH 8.0, 140 mmol/L NaCl, 1 mmol/L EDTA, 10% glycerol, 0.5% NP40, 0.25%. Triton X100). The pellets were then resuspended in washing buffer (10 mmol/L Tris pH 8.0, 1 mmol/L EDTA, 0.5 mmol/L EGTA, 200 mmol/L NaCl), washed and resuspended in shearing buffer (0.1% SDS, 1 mmol/L EDTA, pH 8, 10 mmol/L Tris HCl, pH 8) before sonication using Covaris E220 following manufacturer’s instructions. Chromatin fragments were precipitated using specific antibodies and protein G beads, washed, and treated with proteinase K and RNase A. Purified ChIP DNA was then used for ChIP-qPCR analysis. The forward and reverse primers for ChIP-qPCR are “GCTCCATCCGGGAGACACACAA” and “GCAGGGTCAATGTGCAGTTTCT” respectively.

ChIP-qPCR analysis was performed as described previously [13]. The antibodies used for the RNAPII (Santa Cruz, sc-899); H3K4me1(Abcam, ab8895); H3K4me2 (Abcam, ab32356); H3K4me3 (Abcam, ab8580); H3K27ac (Abcam, ab4729); p300 (Abcam, ab10485); anti-RORγ rabbit serum was generated by Covance, using purified GST-human RORγ fragment (amino acids 79-301) expressed in *Escherichia coli*, SRC-1 (Santa Cruz, sc-8995); SRC-3/ACTR65 and IgG (Santa Cruz, sc-2027). ChIPs were performed with each experimental point in triplicate, and each experiment was repeated three times.

Fastq files from previous datasets [13] were processed by the pipeline of AQUAS Transcription Factor and Histone (https://github.com/kundajelab/chipseq_pipeline). Briefly, sequencing tags were mapped against the reference genome using BWA 0.7.15 [25]. Uniquely mapped tags filtering and deduping were used for peak calling by model-based analysis for ChIP-seq (MACS; 2.1.0) to identify regions of ChIP-seq enrichment over background. Normalized genome-wide signal-coverage tracks from raw-read alignment files were built by MACS2, UCSC tools (bedGraphToBigWig/bedClip; http://hgdownload.cse.ucsc.edu/admin/exe/linux.x86_64/) and bedTools (https://github.com/arq5x/bedtools2). Visualization of ChIP-seq signal at enriched genomic regions (avgprofile and heatmap) was achieved by using deepTools (https://deeptools.readthedocs.io/en/develop/index.html).

### Bioinformatic analyses using clinical dataset

METABRIC data sets were downloaded from cBioPortal website at http://www.cbioportal.org/study?id=brca_metabric#summary. The data were then Log_2_ transformed and quantile normalized before further analysis. Principal component analysis (PCA) was carried out with R ‘COMPADRE’ package [26]. After PCA transformation, the samples were visualized according to pathway activity score using ‘gplots’ R packages. Based on the pathway activity score and the gene profile across the samples, the Pearson correlation metric was computed between each gene by using the ‘cor’ function in R.

### Statistics

Statistical analyses were performed by GraphPad Prism software 7.0. The data are presented as mean values ± SD from at least three independent experiments. Statistical analysis was performed using two-tailed Student’s *t-*tests or ANOVA with Tukey’s post hoc test to compare the means. *P* < 0.05 was considered significant.

## Results

### Time-restricted feeding does not affect cell growth and survival in porcine liver organoids

**Fig. 2:**
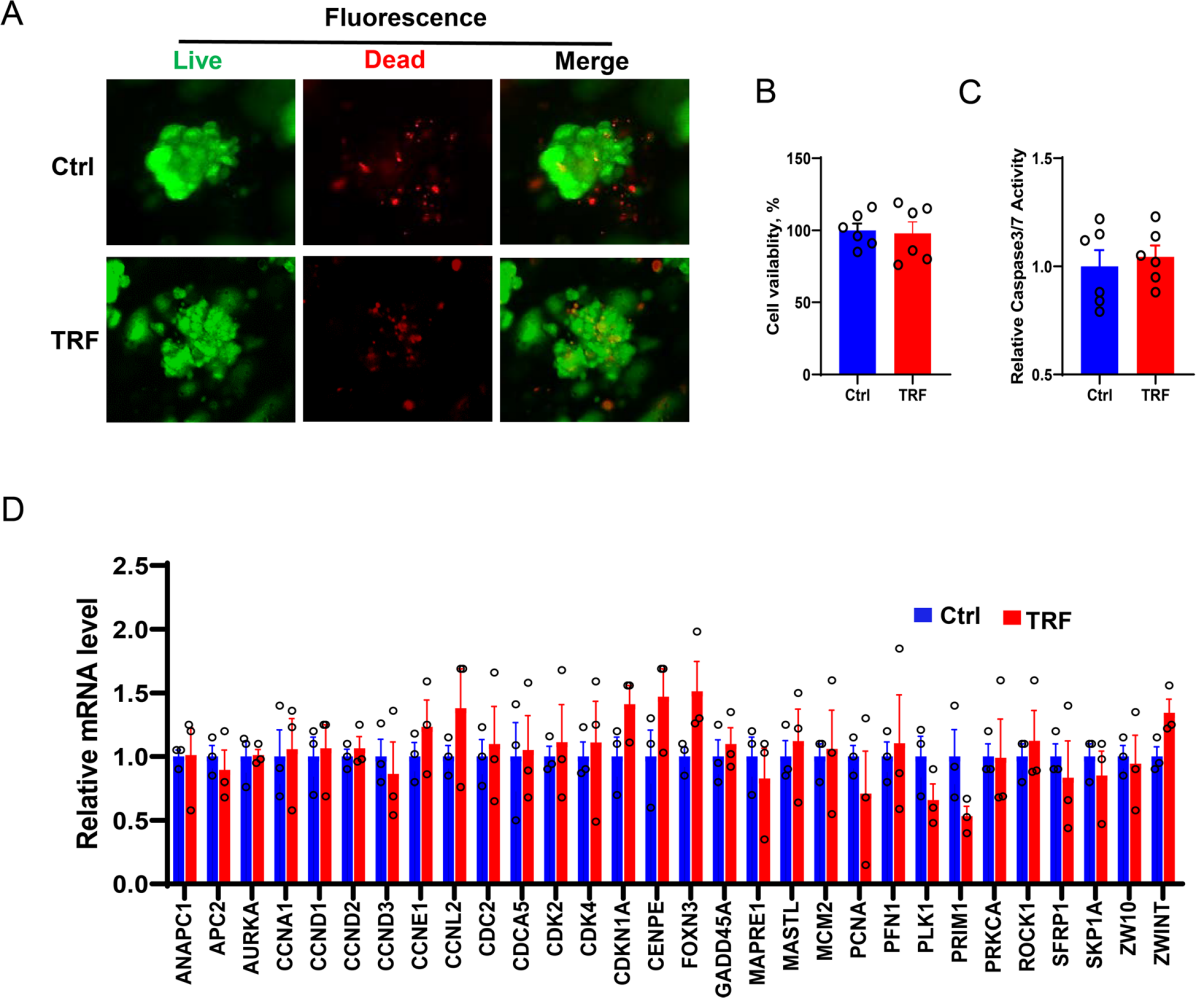
Time-restriction feeding does not affect cell survival and growth in porcine liver organoids. **a** In the established porcine liver organoids model, representative images show the cell viability determined using live/dead reagent staining. **b** The percentage of viable cells (%) quantified by measuring ATP presence, an indicator of metabolically active cells. **c** The cell apoptosis in the porcine liver organoids reflected by the relative caspase 3/7 enzyme activity. **d** The relative mRNA expression of key genes involved in cell cycle/proliferation. Data were presented as means ± SD of at least three independent experiments, *P < 0.05, using two tailed Student's *t*-test-

Given that organoids are more physiologically relevant than 2D monolayers cells, we developed the porcine liver organoids. Firstly, we evaluated the effects of TRF using live/dead regents (calcein AM/ethidium bromide homodimer-1). Immunofluorescent staining showed that TRF did not affect the hepatocytes viability (Fig. 2a), which was further confirmed by quantification of adenosine triphosphate (ATP) presence in cells using a cell-titer measurement, indicative of metabolically active cells (Fig. 2b, *P* > 0.05). In addition, there was no difference of the caspase 3/7 enzyme activity between the control and TRF treated organoids (Fig. 2c, *P* > 0.05). In line with this, TRF had no effects on the key proliferation and survival genes expression in the porcine liver organoids, compared to that in control (Fig. 2d, *P* > 0.05). These data demonstrated that the 10-h TRF regimen does alter hepatocytes physiology significantly in our established porcine liver organoids.

### Time-restricted feeding downregulates cholesterol biosynthesis program

**Fig. 3:**
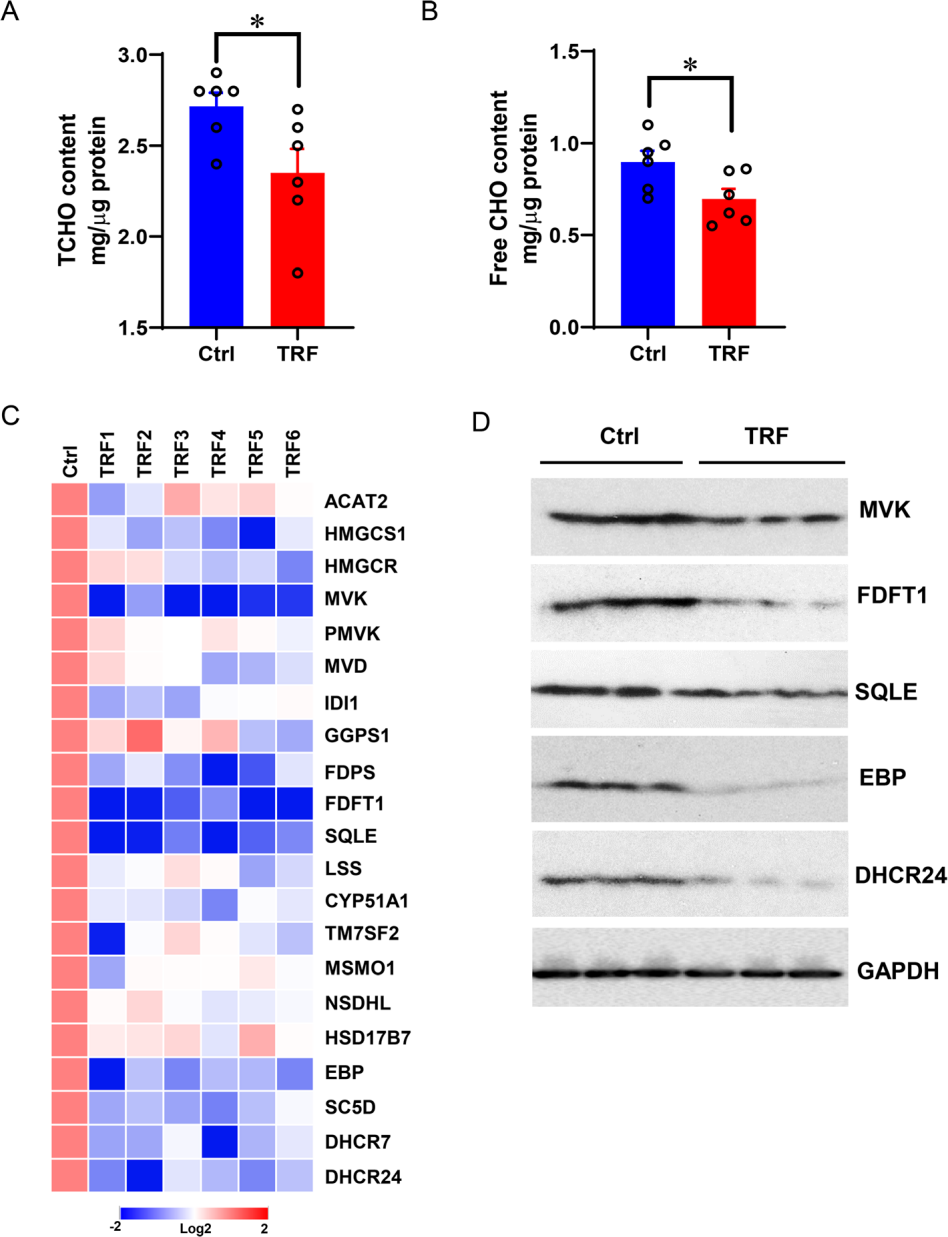
TRF reduces cholesterol biosynthesis program in porcine liver organoids. **a**, **b** Total and free CHO contents in organoids (mg/μg protein). **c** Key genes expression (log_2_) of CHO biosynthesis normalized to control. **d** Western blotting analysis of CHO biosynthesis enzymes MVK, FDFT1, SQLE, EBP, DHCR24 selected based on mRNA expression (**c**), whereas GAPDH was used as an internal reference. Data were presented as means ± SD of at least three independent experiments, *P < 0.05, using two tailed Student's *t*-test

Previous studies have demonstrated that TRF resulted in lower CHO levels in circulation of mice [8, 27]. We thus examined both total and free CHO levels, and showed significantly decreased CHO content in organoids under TRF, compared to the control group (Fig. 3a, b, *P* < 0.05). Furthermore, the expression of key genes involved in CHO biosynthesis were investigated. Consistently, genes such as *MVK* (encoding mevalonate kinase), *FDPS* (encoding farnesyl pyrophosphate synthase), *FDFT1* (encoding farnesyl-diphosphate farnesyltransferase 1), *SQLE* (encoding Squalene monooxygenase), *EBP* (encoding emopamil binding protein), *SC5D* (encoding sterol-c5-desaturase), *DHCR7* (encoding 7-Dehydrocholesterol reductase) and *DHCR24* were significantly downregulated in TRF group (Fig. 3c). In line with the mRNA levels, TRF resulted in strong downregulation of the CHO biosynthesis enzyme proteins including MVK, FDFT1, SQLE, EBP and DHCR24 (Fig. 3d). These results indicated that the CHO biosynthesis program is responsive to TRF treatment.

### RORγ is linked to TRF-induced CHO downregulation

**Fig. 4:**
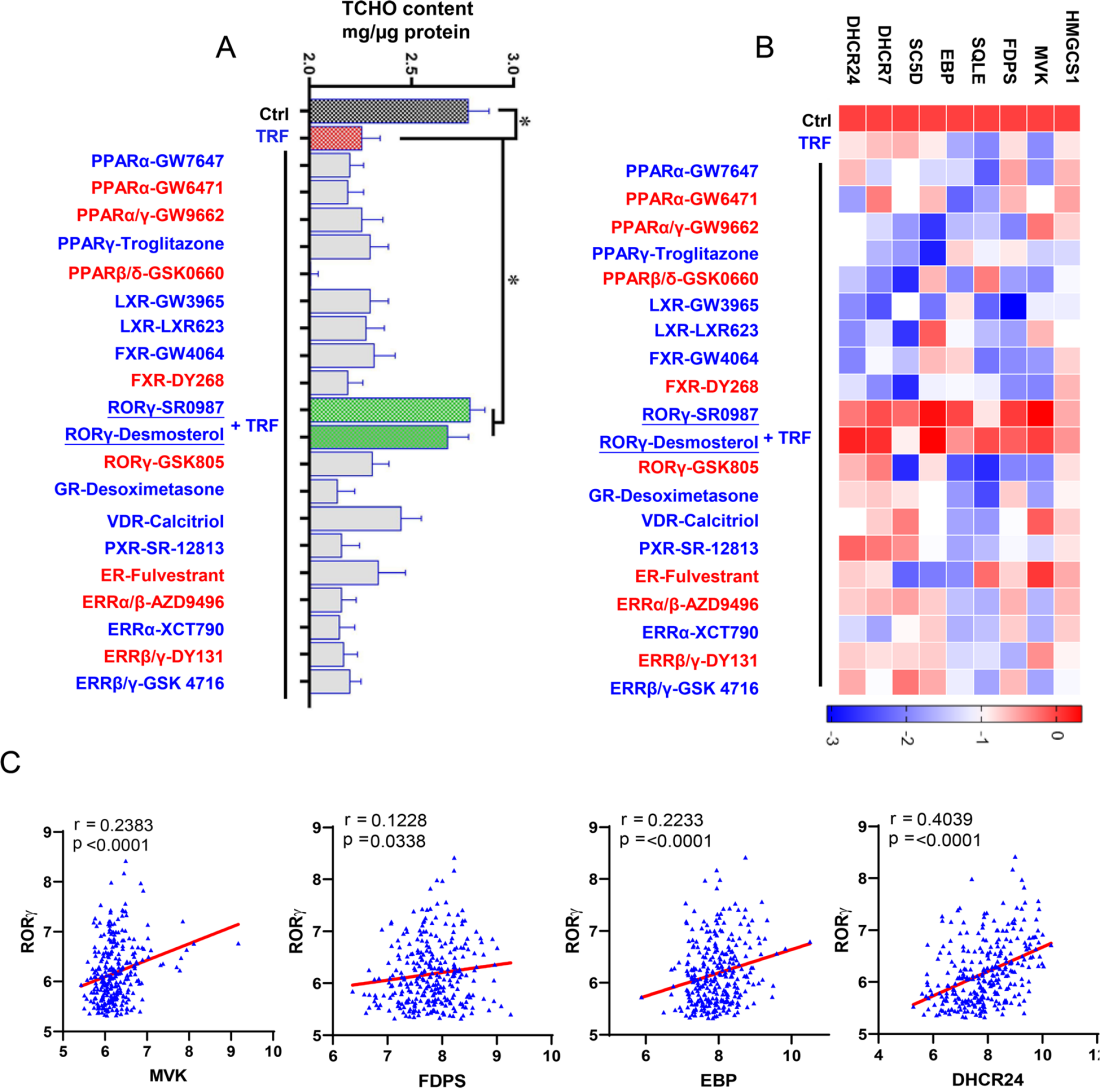
RORγ agonist rescues TRF-induced CHO downregulation. **a** Screening 20 compounds of lipid-related nuclear receptors for the rescue action on TRF-induced lower CHO content (mg/μg protein). **b** In line with the CHO content, SR0987 and desmosterol (RORγ agonists) rescued the CHO biosynthesis gene expression. **c** Pearson correlation analysis based on clinical data revealed that RORγ is positively correlated with CHO biosynthesis genes including *MVK*, *FDPS*, *EBP* and *DHCR24.* Data were presented as means ± SD of at least three independent experiments, *P < 0.05, using two tailed Student's *t*-test. Agonists were in red; antagonists were in blue

Cholesterol biosynthesis pathway is under the tight regulation of major transcription NRs, such as liver X receptors and RORs [13, 28]. To identify potential drivers of the decreased CHO biosynthesis program in TRF treated organoids, we tested a panel of 20 small-molecule modulators targeting members of the NR family in liver organoids (Fig. 4a). Intriguingly, the RORγ agonists SR0987 and desmosterol showed strongest capacity to rescue cellular CHO contents reduction induced by TRF (Fig. 4a, *P* < 0.05). Consistently, we found that the significantly downregulated expression of genes involved in CHO biosynthesis were restored to the levels that comparable to control (Fig. 4b). Furthermore, we analyzed the relevant clinical dataset and revealed a strong positive relationship between the expressions of RORγ gene *RORC* and *MVK* (*r* = 0.2383, *P* < 0.0001), *FDPS* (*r* = 0.1228, *P* = 0.0338), *EBP* (*r* = 0.2233, *P* < 0.0001) and *DHCR24* (*r* = 0.4039, *P* < 0.0001), respectively. Together, these data suggested that RORγ is a key factor linked to the decreased CHO biosynthesis program in TRF treated organoids.

### RORγ is required for TRF-induced CHO biosynthesis program downregulation

**Fig. 5:**
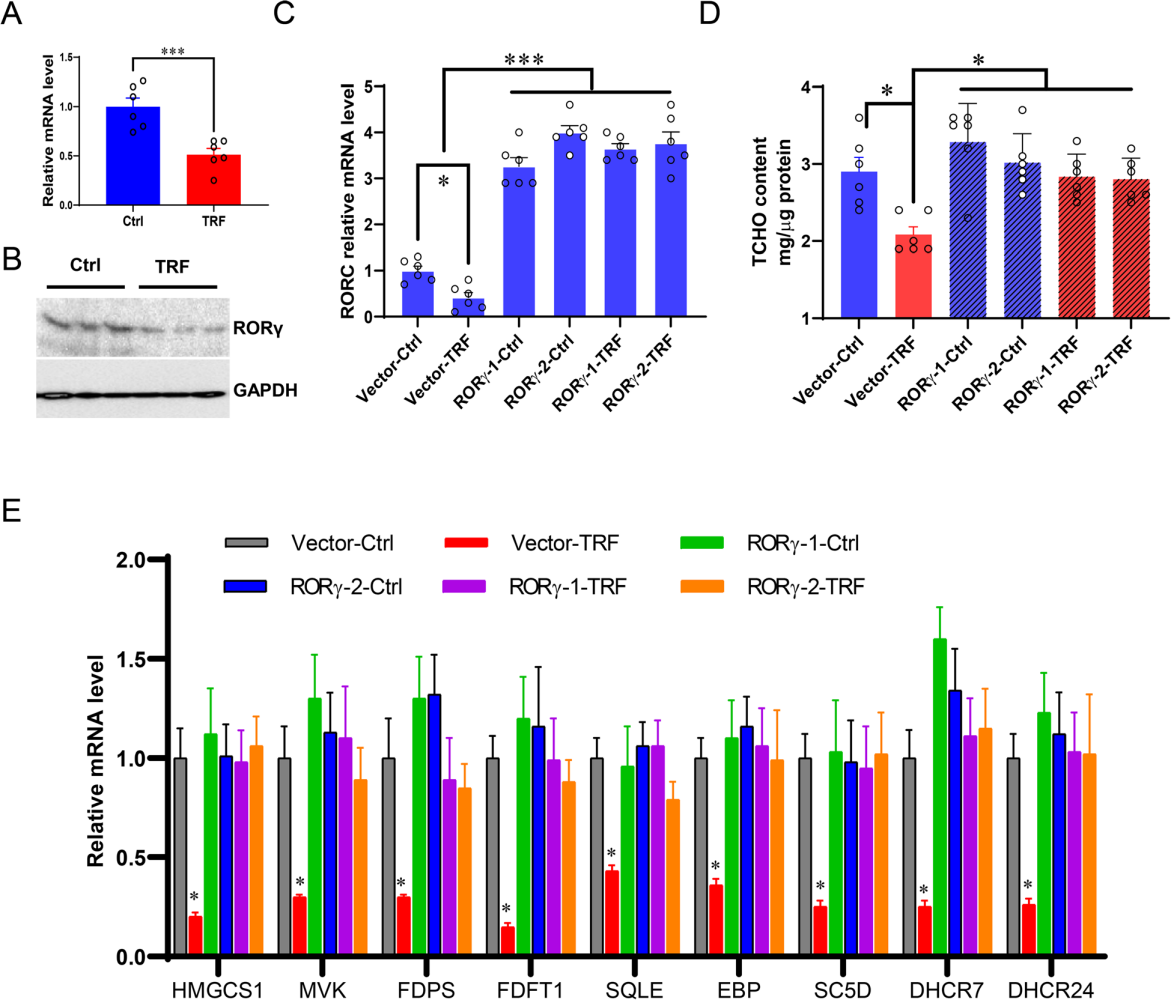
RORγ is linked to TRF-induced CHO downregulation. **a**, **b** Endogenous RORγ mRNA and protein expression in response to TRF. **c** mRNA expression of RORC (gene encoding RORγ) in response to RORγ overexpression with or without TRF treatment (RORγ-1-Ctrl; RORγ-2-Ctrl; RORγ-1-TRF; RORγ-2-TRF), compared to the vector with or without TRF (Vector-Ctrl; Vector-TRF). **d** RORγ overexpression rescued the CHO content (mg/μg protein) in organoids under TRF treatment. **e** RORγ overexpression increased TRF treated organoids CHO biosynthesis genes. Data were presented as means ± SD of at least three independent experiments, *P < 0.05, ***P < 0.001. using two tailed Student's *t*-test or ANOVA with Tukey’s post hoc test

Next, we investigated whether TRF downregulates CHO biosynthesis program via RORγ signaling. First, we examined the endogenous expression of RORγ in porcine liver organoids with qRT-PCR and western blotting, and found that both mRNA (*P* < 0.001) and protein abundances were significantly decreased in TRF treated organoids, when comparing to the controls (Fig. 5a, b). To determine whether elevated RORγ alone is sufficient to promote the CHO biosynthesis program, we overexpressed RORγ in TRF treated organoids and confirmed its high expression compared to vector controls (Fig. 5c, *P* < 0.001). Secondly, as shown in Fig. 5d, ectopic RORγ significantly enhanced the CHO content of TRF treated organoids, but not of the Vector-Ctrl group (*P* < 0.05). Similarly, the key CHO biosynthesis genes were significantly upregulated by overexpressed RORγ, compared to the Vector-TRF group (Fig. 5e, *P* < 0.05). There was a trend that RORγ overexpression in the organoids caused higher expression of these CHO biosynthesis genes than the Vector-Ctrl group, although no statistical significance reached. Together, these results suggested that RORγ plays a direct role in the TRF regulation of CHO biosynthesis program.

### Time-restriction feeding reduces RORγ enrichment on MVK gene promoter

**Fig. 6:**
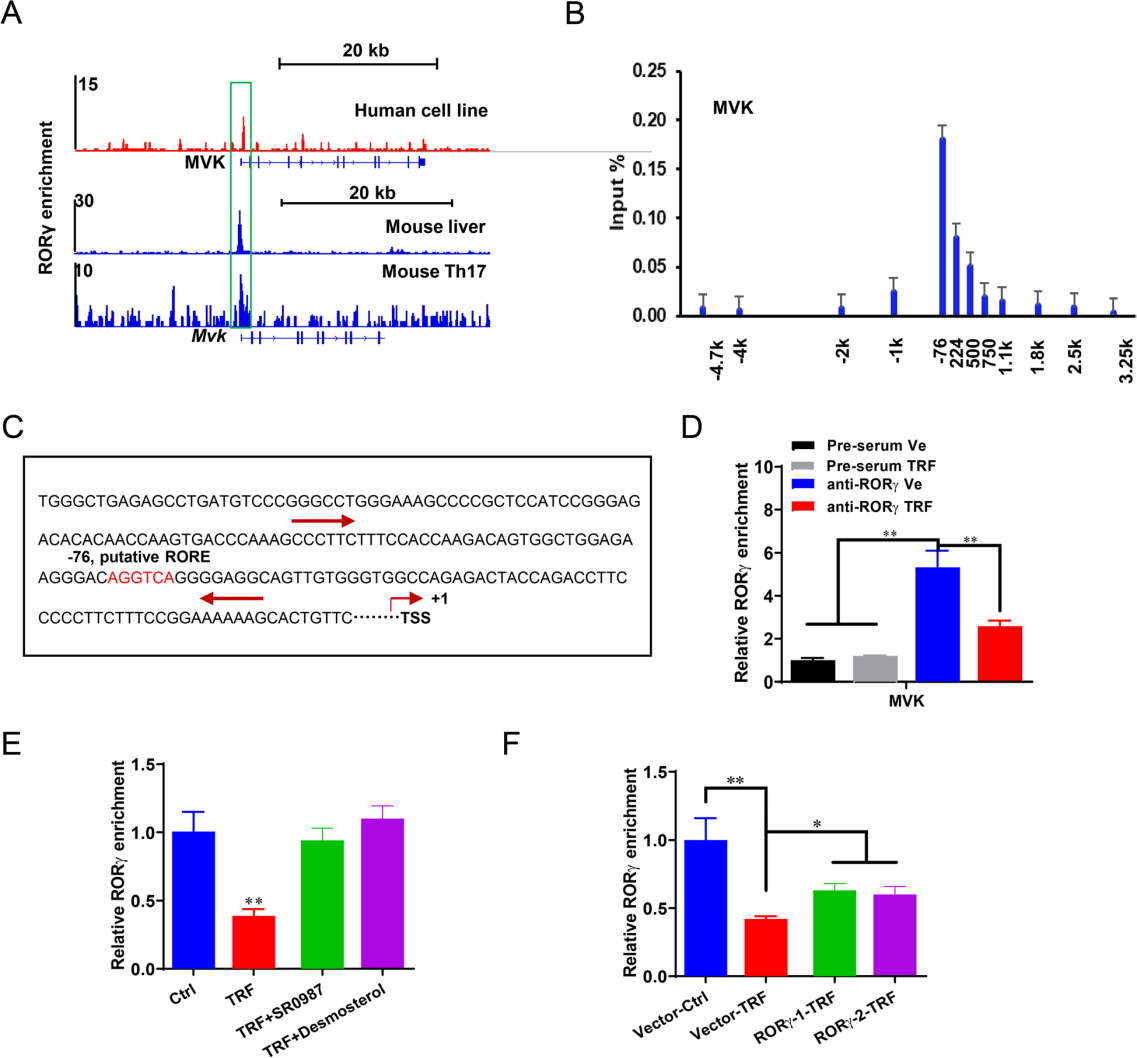
The loss of RORγ binding decreases histone acetylation on CHO genes in TRF treated organoids. **a** RORγ directly binds on CHO gene, MVK, both in human and murine samples analyzed by ChIP-seq datasets. **b** ChIP-qPCR analysis of RORγ occupancy at the locus of MVK in organoids. **c** Schematic diagram depicting the locations of putative ROR element region of MVK gene and primers used for genomic DNA PCR. **d** TRF reduced RORγ enrichment on MVK gene promoter. **e**, **f** RORγ agonists SR0987 and Desmosterol **e** and overexpression (RORγ-1-TRF, RORγ-2-TRF) **f** restored the enrichment of RORγ at target loci of MVK. Data were presented as means ± SD of at least three independent experiments, *P < 0.05, **P < 0.01. using ANOVA with Tukey’s post hoc test

To dissect molecular components of the RORγ pathway in the TRF downregulating CHO biosynthesis program, we examined the impact of TRF on RORγ recruitment to chromatin targets. Firstly, we performed the analysis of an available ChIP-seq data [13, 14, 29], and the results showed that RORγ peaks are present on *MVK* gene both in human (top) and mouse (bottom) (Fig. 6a). It is well-known that the specific sequence motifs of RORγ binding DNA including A(A/T)NTAGGTCA (the classic ROR element motif) or C(T/A)(G/A)GGNCA (the variant RORE motif) [30]. In consistent with the *MVK*-RORγ peak location in human or mouse, ChIP-qPCR of regions containing 12 putative ROREs across the MVK locus demonstrated that RORγ bound to a site around the transcription start site (TSS) region in porcine liver organoids (Fig. 6b). As shown in Fig. 6c, the site contains sequences that match the motif AGGTCA. When organoids were exposed to TRF, RORγ binding was reduced compared to control (Fig. 6d, *P* < 0.01). We next assessed the efficiency of RORγ agonist to restore RORγ binding, SR0987 or desmosterol treatment enhanced 2-fold RORγ occupancy on MVK gene promoter in the TRF treated organoids (Fig. 6e, *P* < 0.01). Interestingly, RORγ enrichment was only increased 50% by RORγ overexpression in TRF treated organoids, compared to the vector-TRF group (Fig. 6f, *P* < 0.05). These data indicated that other factors may also contribute to the RORγ-mediated chromatin modifications in the TRF controlled CHO biosynthesis programming, than RORγ endogenous expression.

### Time-restriction feeding modifies transcription-complex modifications on the loci of RORγ binding

**Fig. 7:**
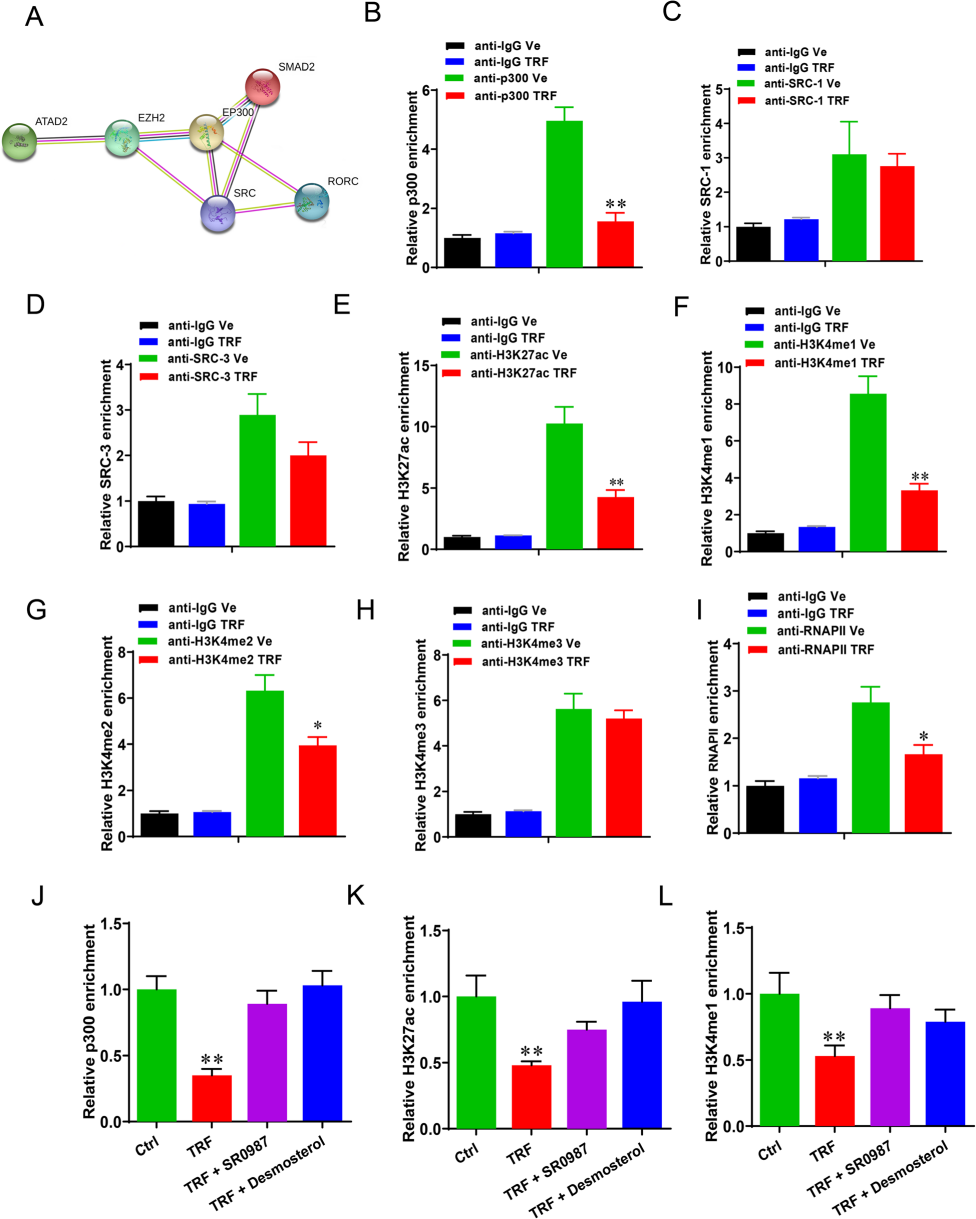
Time-restricted feeding modifies histones modification on the loci of RORγ binding. **a** Putative co-factors of RORγ transcriptional regulation were predicted by STRING. **b**-**d** The relative enrichment of RORγ co-factors (p300, SRC-1 and SRC-3) at the locus of MVK in organoids analyzed by ChIP-qPCR. **e**-**i** The relative enrichment of histone marks (H3K27ac, H3K4me1/2/3) and RNA polymerase II occupancy analyzed by ChIP-qPCR. **j**-**l** RORγ agonists SR0987 and Desmosterol enhanced the occupancies of p300, H3K27ac and H3K4me1 at target loci MVK of TRF treated organoids. Data were presented as means ± SD of at least three independent experiments, *P < 0.05, **P < 0.01. using ANOVA with Tukey’s post hoc test

Next, we investigated whether transcription co-factors or histone modifications facilitated the actions of RORγ in the regulation of CHO biosynthesis program in TRF treated organoids. The putative co-factors p300, SRC-1 and SRC-3 were predicted by STRING analysis from ELIXIR database (Fig. 7a). Of the three factors, only p300 occupancy was significantly reduced on the *MVK* gene in the TRF treated organoids, compared to that of control (Fig. 7b-d, *P* < 0.01). We then performed ChIP-qPCR to detect the transcriptional activation-linked histone marks H3K27ac, H3K4me1/2/3 at the locus of MVK. The results showed that TRF significantly decreased the enrichment of H3K27ac (Fig. 7e, *P* < 0.01), H3K4me1/2 (Fig. 7f, g, *P* < 0.01), but not H3K4me3 (Fig. 7h, *P* > 0.05). In line with the reduction of mRNA levels of CHO biosynthesis genes, promoter occupancies of RNA polymerase II (Pol-II) was also reduced at the target loci in the TRF treated organoids (Fig. 7i, *P* < 0.05). Furthermore, RORγ agonists enhanced the enrichments of p300, H3K27ac and H3K4me1 in the organoids exposed to TRF (Fig. 7j-l, *P* < 0.01). Taken together, these results implied that TRF triggers the RORγ-associated chromatin remodeling at the locus of CHO biosynthesis genes.

## Discussion

Temporal regulation of feeding, i.e. TRF in animal husbandry, may offer a dieting strategy to modify metabolism through the oscillation of hepatic genes expression that are key metabolic regulators [4, 8]. In the current study, we used porcine liver organoids and showed that 10-h TRF regimen does not alter cell viability, proliferation, or apoptosis. Instead, TRF down-regulated hepatic cholesterol biosynthesis program involving MVK, FDFT1, SQLE, EBP and DHCR24 expressions at both mRNA and protein level, associated with the reduced CHO output in the TRF treated organoids. Subsequently, our search for responsible transcription factors focusing on NRs uncovered the predominant role of RORγ. In that RORγ agonist SR0987 and RORγ overexpression reprogrammed the CHO biosynthesis pathway induced by TRF in porcine liver organoids. Finally, we demonstrated that RORγ directly binds to MVK gene, whereas TRF downregulates CHO pathway via RORγ-mediated chromatin remodeling.

Interactions between circadian clock and metabolism can be affected by nutrition quality, quantity or daily eating pattern. Given the growing use of pigs in basic research, as well as in agriculture [18, 31], it is necessary to understand the extent to which circadian rhythms affect this species. By employing a 10-h TRF regimen, we observed a downregulation of CHO biosynthesis program, thus a TRF resultant decreased CHO output in the porcine liver organoids. In accordance, a substantial amount of studies demonstrate that various TRF strategies protect individuals from diet induced obesity and metabolic disorders [6, 10, 32]. For instance, Hatori et al. have shown that 8-h TRF reduced hepatic steatosis and hyperinsulinemia through cAMP-response element binding protein, mTOR (mammalian target of rapamycin) and AMP-activated protein kinase pathways in mice [11]. Recently, 10-h time-restriction eating has been applied to patients diagnosed with metabolic syndrome and showed positive effects including improved body weight, blood pressure and lowered cholesterol levels [10].

Although it is suggested that counteracting hypercholesterolemia is a general hallmark of TRF [6, 33], liver is the master regulator of cholesterol homeostasis of mammals. Our analysis based on clinical data revealed that hepatic expression of MVK, FDPS, EBP and DHCR24 are all positively correlated with the expression of NR family of transcription factor, RORγ. These genes, e.g.*,* MVK encodes mevalonate kinase enzyme, catalyzing the conversion of CHO precursor [34], along with several others that were shown to participate in the TRF-reduced CHO biosynthesis program in our study. The link of CHO genes to RORγ is of great interest, as RORγ is involved in the direct regulation of circadian rhythm by binding to the main clock gene [35]. Studies have revealed that approximately 10% of all liver mRNA are expressed in a rhythmic fashion [8, 36]. We hypothesized that RORγ may represent the dominant respondent of liver oscillator in our TRF-treated porcine liver organoids. Indeed, we found that TRF decreased RORγ expression at both mRNA and protein level in porcine liver. While RORγ agonist rescued the TRF-resulted CHO downregulation, amongst 20 compounds targeting NRs. To support this, it is demonstrated in another study that hepatocyte-specific RORγ knockout mice exhibit improved insulin sensitive due to reduced gluconeogenesis, but also changed lipid metabolic genes [14]. Inversely, we showed that hepatocytes ectopic RORγ disrupted TRF induced-CHO biosynthesis genes downregulation and increased CHO end product in porcine liver organoids, pointing to the critical role of RORγ as the master transcription factor.

It has long been considered that SREBP-2 is the primary transcription factor for activation of genes involved in CHO biosynthesis [37–39], including MVK [40]. In contrast, our previous study has demonstrated that RORγ plays a dominant function over that of SREBP-2 in controlling CHO biosynthesis program in cancerous cells [13], which is in line with our current demonstration. By further examining the downstream events, we identified a RORγ binding site in the DNA sequence of MVK in porcine liver organoids. We have shown clearly that TRF reduces RORγ enrichment at the locus of MVK, involving the reduced enrichments of co-factor p300 and histone marks H3K27ac and H3K4me1/2. While RORγ agonists enhanced the occupancies of p300, H3K27ac and H3K4me1 at target loci against TRF regulation. We therefore suggested that RORγ is a targetable master regulator of CHO biosynthesis program during the temporal regulation of feeding and beyond.

## Conclusions

In conclusion, we identified a novel connection between the regulator RORγ and the temporal regulation of hepatic CHO biosynthesis program in porcine organoids. Our findings showed the potential of organoids to be used as a platform for mechanistic studies and drug testing. More importantly, we contributed to the development of an optimal long-term organ culture and its application to animal husbandry. Challenges remain as to capture complex pathologies of liver diseases in a dish, such as inflammation and fibrosis [41], and further studies are warranted.

## Supplementary information

**Additional file 1: Table S1.** Nucleotide sequences of specific primers used for real-time PCR.

**Additional file 2: Table S2.** Antibodies used.

## Data Availability

The datasets analyzed during the current study are available from the corresponding author upon request.

## Abbreviations

ATP: Adenosine triphosphate
CHO: Cholesterol
DEX: Dexamethasone
DHCR7: 7-Dehydrocholesterol reductase
EBP: Emopamil binding protein
FDFT1: Farnesyl-diphosphate farnesyltransferase 1
FDPS: Farnesyl pyrophosphate synthase
mTOR: Mammalian target of rapamycin
MVK: Mevalonate kinase
NAFLD: Nonalcoholic fatty liver disease
NRs: Nuclear receptors
PCA: Principal component analysis
Pol-II: Polymerase II
ROCK: Rho kinase
ROR: Retinoic acid-related (RAR)-related orphan receptor
RORE: ROR element
SC5D: Sterol-C5-desaturase
SQLE: Squalene monooxygenase
SREBP-2: Sterol regulatory element-binding protein-2
TRF: Time-restricted feeding
TSS: Transcription start site
