# Supplementary material for: Time-restricted feeding downregulates cholesterol biosynthesis program via RORγ-mediated chromatin modification in porcine liver organoids
Source: J Anim Sci Biotechnol. 2020 Nov 2;11:106. doi: 10.1186/s40104-020-00511-9 (PMC7604961; doi:10.1186/s40104-020-00511-9)
Supplement: Supplementary file 1 — Additional file 1: Table S1. Nucleotide sequences of specific primers used for real-time PCR. [file 40104_2020_511_MOESM1_ESM.doc]

**Table S1.** Nucleotide sequences of specific primers used for real-time PCR

| Target genes | Primer sequences (5′ to 3′) | |
| --- | --- | --- |
| *ANAPC1* | Forward: | ACCCAACACTTGTCAGAACCACTG |
|  | Reverse: | GTGGCATTCCTTCACCCTTCAGAC |
| *APC2* | Forward: | CCACCAAGCCGCAGCAAGAC |
|  | Reverse: | GGCAGTCAGCAATGTCCAGGAAG |
| *AURKA* | Forward: | AGCCAGGGACCTCATCTCAAGAC |
|  | Reverse: | TTAGGGTTGCTTGCTGGTGGATTC |
| *CCNA1* | Forward: | AGGAAGGACTGCGGGTGGTTC |
|  | Reverse: | ATTGTGCCTTGCCTGAGTGAGC |
| *CCND1* | Forward: | CACCGCTTCCGCTTACCTCAAC |
|  | Reverse: | GCCTTGGAGCACACCTGTCAC |
| *CCND2* | Forward: | CCGACAACTCCATCAAGCCTCAG |
|  | Reverse: | CAGCCGCCAGGTTCCACTTC |
| *CCND3* | Forward: | GCGGAAGATGCTGGCGTACTG |
|  | Reverse: | CAGGACAGGTAGCGATCCAGGTAG |
| *CCNE1* | Forward: | CGAGTGCTCCAGATGCTGCTAAG |
|  | Reverse: | TGCTCCGCTTAATACGACACAACC |
| *CCNL2* | Forward: | AGAGTGAGGTGGAGAAGCGTAGG |
|  | Reverse: | CCTGGCATTCTTCGCAGAGAGC |
| *CDC2* | Forward: | AACCACTTTTCCACGGGGATTCAG |
|  | Reverse: | GCTAGGCTTCCTGGTTTCCACTTG |
| *CDCA5* | Forward: | AGCAGAAGCCAGAAGACCCTCAG |
|  | Reverse: | GCCGTGCTCAGGAGTGTAAACAG |
| *CDK2* | Forward: | CCAACAAGCGGATTTCAGCAAAGG |
|  | Reverse: | AGGGTGAGACGAGGGGTAGGG |
| *CDK4* | Forward: | CGGAGATTGGTGTTGGTGCCTATG |
|  | Reverse: | CCATTGGGGACTCTTACGCTCTTG |
| *CDKN1A* | Forward: | GGACCATGTGGACCTGTTGCTG |
|  | Reverse: | TTTCCTGCCCTGGGAGGTGTC |
| *CENPE* | Forward: | CAAGATCCTGGGCCAAAGGAATCG |
|  | Reverse: | CACTCGGGCACGTCTTCTTCC |
| *FOXN3* | Forward: | GCGACTGTGTCTCCTCCTCCTC |
|  | Reverse: | ACTGTGGCTCTCGTGGCTCTG |
| *GADD45A* | Forward: | CCTTTGTCCTTCAGTGGCTGTGAG |
|  | Reverse: | ATCGCTCGCTGGCCTCCTTAG |
| *MAPRE1* | Forward: | TCTGCTCCCCTTGCCCACTG |
|  | Reverse: | CACCTGCCTGCTGAACTTCCAC |
| *MASTL* | Forward: | AGACCTCCTGCCTACATCGC |
|  | Reverse: | TGGACGGTTCTGAGCTGGTG |
| *MCM2* | Forward: | TACGACAGTGATGAGGAGGAGGAG |
|  | Reverse: | AGGAAGTTCTTGAAGCGGTGGTG |
| *PCNA* | Forward: | TGTAGCCGCGTCGTTGTGATTC |
|  | Reverse: | CGCTTCCAGCACCTTCTTCAGG |
| *PFN1* | Forward: | CACCATGACTGCCAAGACGCTAG |
|  | Reverse: | GGAGTGAAGGGGAGGGACAGAC |
| *PLK1* | Forward: | CCTGGAGGAGTTCGGCTGCTC |
|  | Reverse: | TGGTGGTGGTGGTGGTGGAG |
| *PRIM1* | Forward: | CGTGCTAATCCTGCCTGGGTTG |
|  | Reverse: | TTTACCACTCCGCCATAGTTCAGC |
| *PRKCA* | Forward: | ACCAAGACCATCCGCTCTACACTC |
|  | Reverse: | TCGGTCCCAGTCCCAGATTTCTAC |
| *ROCK1* | Forward: | AGAGCAGCAAAAGTGGGTTAGTCG |
|  | Reverse: | CTAGGAGATGACCGTGCAAAAGGG |
| *SFRP1* | Forward: | GCGAGTTTGCCCTGAGGATGAAG |
|  | Reverse: | CATGATGAGGAAGTGGTGGCTGAG |
| *SKP1A* | Forward: | TACCCACCACAAGGATGACC |
|  | Reverse: | ATGTCTAGGTAGTTTGCTGCCA |
| *ZW10* | Forward: | CTGCCATTCACCACAACAACTGC |
|  | Reverse: | ACTCTGTCCCAAGTCTCCTGAAGC |
| *ZWINT* | Forward: | AGAACTGCCCGCCCAGATCC |
|  | Reverse: | TCAGAAGCCAAGGGGTCCAAGC |
| *ACAT2* | Forward: | TAATGATGGTGCTGCTGCTGTGG |
|  | Reverse: | GCTTGCTTTATTGCCGGGATTGG |
| *HMGCS1* | Forward: | AAGCACAGCCACCGAGCATATTC |
|  | Reverse: | ACCATCCCACCCCACACTGAAG |
| *HMGCR* | Forward: | TGTGATTGGAGTTGGCACCATGTC |
|  | Reverse: | ACACGCAAGCTGGGAAGAAAGTC |
| *MVK* | Forward: | GTTGTCTCAAGTCCTGCTGGTGTC |
|  | Reverse: | AGGCTCACTTTCCCACTGTTGTG |
| *PMVK* | Forward: | GGTGGATGATGCTGAGTCAGAGTG |
|  | Reverse: | GTGCTGCTCATCTCCGTGGTTC |
| *MVD* | Forward: | GCCACCTGCTTGGACACCTTC |
|  | Reverse: | GGCGAAGATCACGGCGTTGG |
| *IDI1* | Forward: | TGCTCCAACAACGATCAGATGCC |
|  | Reverse: | TTAAACGCCTCTGTGCTGCTCTTC |
| *GGPS1* | Forward: | GTGGCACACAGCATCTATGGAA |
|  | Reverse: | GCCTTGGCCCTGATGTAGTT |
| *FDPS* | Forward: | TGGCAGACGACATCGAGGACTC |
|  | Reverse: | CTTCAGCAGGCGGTAGATACAAGC |
| *FDFT1* | Forward: | GCGTCCACCCTCCTCACTCC |
|  | Reverse: | CCCACACAGCCAGAGCCAAAG |
| *SQLE* | Forward: | TGTGGACCTTTCTCGGCATTGC |
|  | Reverse: | TAGCGACAGCGGTAGGACAGC |
| *LSS* | Forward: | GAGGACCCGCTGGTCCA |
|  | Reverse: | CCACACTGTTCCTGTGCGC |
| *CYP51A1* | Forward: | GCAGGTAACTGGCGTCAATCTCC |
|  | Reverse: | CGATGGCTTGGCGGAACAGG |
| *TM7SF2* | Forward: | ACCCACGCATCTGTTCCTTTGAC |
|  | Reverse: | GGAAGCCATTGACCAGCCACATG |
| *MSMO1* | Forward: | CCTGGGTGACCGTTCGTTTGATAG |
|  | Reverse: | GGTGGAAGTCATGGTGACGAGAAC |
| *NSDHL* | Forward: | TTTGTGATCGGGAACGGGAAGAAC |
|  | Reverse: | TTCGTCATTGGTGATGTGGAAGGC |
| *HSD17B7* | Forward: | GAGGACATCCAGCACAGCAAAGG |
|  | Reverse: | CGGTTCAAAGCCACACTCACAAAG |
| *EBP* | Forward: | TCCTGACAGAGCACCGTGACG |
|  | Reverse: | CAGCACCAGCCACAGAGCATTC |
| *SC5D* | Forward: | ACCCTCTGGATGGCTTCCTTCAG |
|  | Reverse: | AGCTCTGGGGAACACGGAAGTC |
| *DHCR7* | Forward: | AGTCTCGGCAGTGTCAGCAATG |
|  | Reverse: | CTGGTCGCAGGCCATGATGAAG |
| *DHCR24* | Forward: | CCTCTTCCTCCTGCCGCTCTC |
|  | Reverse: | TGCCCTGCTCCTTCCATTCCC |
| *RORC* | Forward: | CAATGGAAGTGGTGCTGGTCAGG |
|  | Reverse: | GGGAGCGGGAGAAGTCAAAGATG |
| ChIP-PCR primers | | |
| MVK-RORE | Forward: | GCTCCATCCGGGAGACACACAA |
|  | Reverse: | GCAGGGTCAATGTGCAGTTTCT |
