# Supplementary material for: Time-restricted feeding downregulates cholesterol biosynthesis program via RORγ-mediated chromatin modification in porcine liver organoids
Source: J Anim Sci Biotechnol. 2020 Nov 2;11:106. doi: 10.1186/s40104-020-00511-9 (PMC7604961; doi:10.1186/s40104-020-00511-9)
Supplement: Supplementary file 2 — Additional file 2: Table S2. Antibodies used. [file 40104_2020_511_MOESM2_ESM.docx]

**Table S2.** Antibodies used.

| Antibody | Vendor | Catalog number | Dilution |
| --- | --- | --- | --- |
| RORγ | eBioscience | 14-6988-82 | 1:1000 |
| MVK | Protein tech | 12228-1-AP | 1:1000 |
| FDFT1 | Santa Cruz | sc-271602 | 1:1000 |
| SQLE | Santa Cruz | sc-271651 | 1:1000 |
| EBP | Santa Cruz | sc-374267 | 1:1000 |
| DHCR24 | Santa Cruz | sc-398938 | 1:1000 |
| GAPDH | Cell signaling | #2118 | 1:3000 |
